# A novel eurythermic and thermostale lipase LipM from *Pseudomonas moraviensis* M9 and its application in the partial hydrolysis of algal oil

**DOI:** 10.1186/s12896-015-0214-0

**Published:** 2015-10-14

**Authors:** Wenjuan Yang, Hai Cao, Li Xu, Houjin Zhang, Yunjun Yan

**Affiliations:** Key Laboratory of Molecular Biophysics of the Ministry of Education, College of Life Science and Technology, Huazhong University of Science and Technology, Wuhan, 430074 P. R. China

**Keywords:** *Pseudomonas moraviensis*, Lipase, Eurythermic, Thermostability, Hydrolysis of algal oil

## Abstract

**Background:**

Lipases are regularly used in biotechnology to catalyse the hydrolysis of triglycerides and the synthesis of esters. Microbial lipases in particular have been widely used in a variety of industrial applications. However, the current commercial microbial lipases cannot meet industrial demand due to rapid inactivation under harsh conditions. Therefore, in order to identify more stable enzymes, we isolated novel eurythermic and thermostable lipase(s) from *Pseudomonas moraviensis* M9.

**Methods:**

Cloning of lipM was based on Touchdown PCR and genome walking, and then recombinant LipM was purified by guanidine hydrochloride and the nickel-nitrilotriacetic acid resins affinity chromatography. Finally, the hydrolysis of algal oil by LipM was analyzed by gas chromatograph-mass spectrometer, thin layer chromatography and gas chromatograph.

**Results:**

The *lipM* gene was first cloned from *Pseudomonas moraviensis* M9 via Touchdown PCR and genome walking. Sequence analysis reveals that LipM is a member of subfamily I.3 of lipases, and the predicted amino acid sequences of LipM has 82 % identity to lipase LipT from *Pseudomonas mandelii* JR-1, and 54 % identity to lipase PML from *Pseudomonas* sp. MIS38 and lipase Lip I.3 from *Pseudomonas* sp. CR-611. LipM was expressed in *Escherichia coli*, purified from inclusion bodies, and further biochemically characterized. Purified LipM differed significantly from previously reported subfamily I.3 lipases, and was eurythermic between 10 °C–95 °C. LipM activity was enhanced by Ca^2+^, Sr^2+^, Mn^2+^, and Ba^2+^, but sharply inhibited by Cu^2+^, Zn^2+^, Co^2+^, Ni^2+^, and EDTA. Compared with other lipases, LipM exhibited medium tolerance to methanol, ethanol, and isopropanol. When applied for hydrolysis of algal oil, LipM could enrich 65.88 % polyunsaturated fatty acids, which include 1.25 % eicosapentaenoic acid, 17.61 % docosapentaenoic acid, and 47.02 % docosahexaenoic acid with derivative glycerides containing 32.46 % diacylglycerols.

**Conclusions:**

A novel eurythermic I.3 subfamily lipase with high tolerance and stability was identified from *Pseudomonas moraviensis* and biochemically characterized. It will not only improve our understanding of subfamily I.3 lipases, but also provides an ideal biocatalyst for the enrichment of polyunsaturated fatty acids. *Pseudomonas moraviensis* have been investigated as a potential resource of lipases.

## Background

Lipases (EC 3.1.1.3), known as triacylglycerol acylhydrolases, are capable of catalyzing hydrolysis of long chain triacylglycerides into free fatty acids and glycerols in aqueous solutions and conducting synthetic reactions in organic media [1]. Lipases widely exist in animals, plants and microorganisms. So far, the majority of microbial lipases characterized are from bacteria and fungi [2]. Compared with fungal lipases, bacterial lipases are generally produced in higher yield and with lower production costs, are more amenable to genetic manipulation, exhibit improved stability in organic solvents, and possess a more diverse range of catalytic activities and substrate specificities [3]. Therefore, bacterial lipases have received a great deal of attention, especially in biotechnological industries such as the food, detergents, oil manufacture, fine chemicals, biodiesel, optically active drugs, and polymeric materials [4].

For most lipase-catalyzed reactions in industrial applications, it is important that lipases remain active in extreme conditions, such as elevated temperatures, organic solvents and/or detergents [1, 5]. Assuming these criteria are met, lipases may one day completely replace their chemical counterparts in industrial applications and green manufacturing processes [2]. Unfortunately, the chemical modifications that occur in such harsh conditions may lead to inactivation of the lipases and hinder their large-scale application in industry [6]. Therefore, lipases resistant to chemical denaturation are highly desirable, and bacterial lipases are the logical choice [7]. Frustratingly, despite rapid development of biochemical engineering, bacterial lipases isolated from *Bacillus* [4, 7], *Burkholderia* [2, 8] and those identified in metagenomic libraries [3, 9] have thus far failed to fulfill industrial applications, due to poor tolerance of high temperatures. For example, lipases from *Pseudomonas* are usually mesophilic [10, 11] or psychrophilic [12]. Meanwhile, lipases from bacteria and archaea tolerating high or low temperatures, extremes of pH or high concentrations of salts, the so-called extremophiles, have special enzymatic characteristics, which may meet the demands of various industrial applications. However, the available number is very limited [13]. Thus, isolating eurythermic bacterial lipases is a high priority.

Polyunsaturated fatty acids (PUFAs) such as eicosapentaenoic acid (EPA), docosapentaenoic acid (DPA) and docosahexaenoic acid (DHA) from functional oils (of alga or fish origin) are used to prevent and treat cancers, arteriosclerosis, inflammation and hyperlipidemia [14]. However, the contents of PUFAs of natural resources are usually less than the standard level of market product, which cannot well meet necessary intake of people [14–16]. Therefore, in recent years, studies on enrichment of PUFAs have been conducted via using fungal lipases [15–19], but most fungi lipases are sensitive to harsh conditions. On the other hand, existing bacterial lipases possessing such activity lack fatty acid selectivity [20, 21]. Hence, investigations for novel bacterial lipases with efficient enrichment of PUFAs are urgently needed.

*P. moraviensis* M9, isolated from Xinjiang Autonomous Regions of China, exhibited a clear degradation halo when grown on M9 medium containing olive-rhodamine B. But, lipases from this species have not yet been deposited in the Lipase Engineering Database (http://www.led.uni-stuttgart.de/). Therefore, in the present study, we successfully cloned the novel subfamily I.3 lipase *lipM* from *P. moraviensis* M9 genomic DNA via touchdown PCR and genome walking, expressed the enzyme in *Escherichia coli*, purified by refolding from inclusion bodies, performed biochemical properties characterization and investigated its use in the hydrolysis of algal oils to enrich PUFAs.

## Methods

### Bacterial strains, plasmids and chemicals

*Pseudomonas moraviensis* M9 isolated from soil samples of Xinjiang Autonomous Regions of China was preserved in China Center for Type Culture Collection (CCTCC), College of Life Sciences of Wuhan University, Wuhan, China, with a strain preservation number of CCTCC AB 205292. The strain M9 grew at 37 °C in Luria-Bertani (LB) broth or on agar plates.* E*. *coli* strains DH5α and BL21 (DE3) (Novagen, Germany) were maintained at 37 °C in LB broth or on agar plates for recombinant plasmid amplification and protein heterologous overexpression. The vector pET-22b (+) (Novagen, USA) was used for gene expression. Genome walking kits, restriction endonucleases, T4 DNA ligase, and Taq DNA polymerase were all got from TaKaRa (Japan). p-Nitrophenyl (p-NP) esters were bought from Sigma-Aldrich (USA). All other chemicals used were of analytical grade and were commercially available from Sinopharm Chemical Reagent Co., Ltd (Shanghai, China).

### Cloning of lipM by Touchdown PCR and genome walking, and sequence and structure analysis

All used primers are listed in Table 1. Degenerate primer design was conducted using CODEHOP (http://blocks.fhcrc.org/codehop.html). A partial lipase sequence was amplified from *P*. *moraviensis* M9 genomic DNA by touchdown PCR [22] using degenerate primer T5 and T3. To obtain the upstream and downstream sequences of the partial lipase gene, a genome walking PCR was performed using a genome walking kit according to the manufacturer’s instructions.

**Table 1:** Primers used for gene cloning and expression

Primers	Function	Sequence (5'-3')
T5	Degenerate primer	ACAATGTGCTGAACATNGGNTAYGARA
T3	Degenerate primer	GAAGGCGCCGCTGAANANNWANGTNTT
lipM9-5-1	Upstream genome walking	GACGCTTCCGCCAGGTTG
lipM9-5-2	Upstream genome walking	AGCCGTGAGCCGACCAGTTC
lipM9-5-3	Upstream genome walking	CCTGGTGTCCGTCGTGCTTG
lipM9-3-1	Downstream genome walking	GCTGTACGTGCGTGATGCCTAT
lipM9-3-2	Downstream genome walking	GGTGGGGGAGCAAGGAGGT
lipM9-3-3	Downstream genome walking	ACGGCAACACACTGGCAGC
lipM9-F	Complete genetic sequence	ATGGGAYTGTTCGATTACARAAAYGCCGA
lipM9-R	Complete genetic sequence	TYASGCRAASRYGAWRCYYGAAKCCGACAGG
lipM9-NF	Expression vector construction	CT*CATATG*GGATTGTTCGATTAC
lipM9-XR	Expression vector construction	CT*CTCGAG*CGCAAACATGATACTT

a): Note: N represents A, C, G, or T; Y identifies C or T; R represents A or G; W identifies A or T. b): *Nde* I, *Xho* I restriction sites in primers are underlined, respectively

Sequence alignments of the DNA and protein sequences were carried out using blastn and blastp, respectively (http://www.ncbi.nlm.nih.gov/BLAST/). Multiple sequence alignment was conducted using Clustal W2 (http://www.ebi.ac.uk/Tools/msa/Clustalw2/) and presented using ESPript 2.2 (http://espript.ibcp.fr/ESPript/ESPript/). Phylogenetic analysis was done with MEGA 5.0 using neighbor-joining method. A bootstrap analysis with 1000 replicates was used to estimate the reliability of the tree [9]. The ExPASy proteomics server (http://us.expasy.org/tools/protparam.html) was used to analyze the protein physicochemical parameters (ProtParam tool). Signal peptide was predicted using the SignalP 4.1 server (http://www.cbs.dtu.dk/services/SignalP/). The 3D structure of the target protein LipM was constructed by SWISS-MODEL (http://swissmodel.expasy.org/) using reported 3D structures of Pseudomonas sp. MIS38 lipase (PDB: 2z8x) as templates and presented in PyMOL.

### Expression and purification of the recombinant LipM

The PCR product amplified by primers lipM9-NF and lipM9-XR was inserted into pET-22b (+) digested with *Nde* I and *Xho* I. The recombinant plasmid pET-22b-*lipM* was transformed into E. coli BL21 (DE3) cells. Transformed cells were inoculated at a dilution of 1:100 in each 1000 ml fresh LB medium containing ampicillin and grown aerobically at 37 °C. The recombinant protein LipM was induced with 0.2 mM IPTG at 16 °C for 20 h after the OD_600_ reached 0.6.

LipM was purified from* E*. *coli* cell extracts as per a previously described method for inclusion body solubilization of lipase PML from *Pseudomonas* sp. MIS38 [10] with minor modification. The cells were harvested by centrifugation and resuspended in lysis buffer (50 mM Tris–HCl buffer, 50 mM NaCl, 1 mM EDTA, pH 8.0), then disrupted by One Shot Cell Disrupter (Constant Systems, British). After centrifugation, the precipitate was washed with lysis buffer containing 2 % Triton X-100, and then dissolved in the buffer (50 mM Tris–HCl, 1 mM EDTA, 5 % glycerol, 10 mM DTT and 6 mM guanidine hydrochloride) overnight at 4 °C. The insoluble components were removed by centrifugation at 8,000 g for 30 min at 4 °C. The collected solubilized inclusion bodies were step-by-step dialyzed against refolding buffer (20 mM Tris–HCl, 10 mM CaCl_2_, pH 8.0) containing 6 mM, 4 mM, 2 mM, 0 mM guanidine hydrochloride (GuHCl), respectively. Finally, the dialyzed solution was applied to the nickel-nitrilotriacetic acid (Ni-NTA) resins affinity chromatography column (GE Healthcare, USA) that had been previously equilibrated with washing buffer (20 mM Tris–HCl pH 8.0, 0.5 mM NaCl, 10 mM CaCl_2_). Then, the target recombinant enzymes were eluted using an imidazole concentration gradient (0, 30, 60, 100, and 200 mM) washing buffer.

The purified LipM was analyzed by 12 % sodium dodecyl sulphate-polyacrylamide gel electrophoresis (SDS-PAGE) stained with Coomassie brilliant blue R-250. Protein concentration was estimated spectrophotometrically according to the method of Bradford using BSA as standard [4]. The single protein band after purification was confirmed by the peptide mass fingerprinting (Yanxing, China). Additionally, for Western blot analysis, proteins were transferred from SDS-PAGE onto polyvinylidene fluoride (PVDF) membrane. The membrane was blocked with 5 % milk, followed by incubation with anti-His tag (1:2000) primary antibody, and then with horseradish peroxidase (HRP)-conjugated goat anti-mouse IgG second antibody (Tiangen, China) as described by the manufacturer. Finally, the blot was detected by Diaminobenzidine (DAB) coloration.

### Characterization of LipM

All *p*-NP esters (acetate C2, butyrate C4, caprylate C8, decanoate C10, laurate C12, myristate C14, and palmitate C16) were respectively dissolved in anhydrous acetonitrile [23] at a concentration of 100 mM as substrates for analysis of substrate specificity. In a standard assay, the total reaction system of 1ml contains 940 μl of Tris–HCl buffer (50 mM, pH 8.0), 10 μl of *p*-NP ester (100 mM), 40 μl of ethanol and 10 μl of the diluted enzyme solution [22]. The blank contained the same components except enzyme solution. Unless otherwise described, lipase activity was measured by this standard assay at 65 °C for 10 min. All experiments were performed in triplicates. One unit of lipase activity (U) was defined as the amount of enzyme that released 1 μmol *p*-NP per minute under assay condition. In addition, activity of the purified lipase was also measured by titration of free fatty acids released by the hydrolysis of olive oil using the pH state method [24]. The measurements were conducted in triplicates. One unit was defined as the amount of enzyme liberating 1 μmol of fatty acid per minute.

The effect of pH on LipM was investigated at 65 °C in buffers with pH ranging from 4.0 to 10.0: citrate-phosphate buffer (pH 4.0-6.5), Tris–HCl buffer (pH 7.0-8.5), and glycine–NaOH buffer (pH 9.0-10.0). The optimal temperature was detected at 0-100 °C with an interval of 5 °C (except 0 °C and 10 °C) in Tris–HCl buffer (pH 8.0) using *p*-NP caprylate as substrate. To determine the thermal stability of LipM, the purified enzyme was incubated in 50 mM Tris–HCl (pH 8.0) at various temperatures, ranging from 60 to 85 °C for different time intervals from 0 to 12 h. At each time interval, samples were pipetted out and the residual activity was immediately assayed at 65 °C and pH 8.0 for 10 min using *p*-NP caprylate as substrate. The kinetic parameters of LipM were tested in 50 mM Tris–HCl (pH 8.0) at 65 °C using* p*-NP caprylate at different concentrations (0.01, 0.02, 0.05, 0.1, 0.2, 0.4, 0.8, 1 and 2 mM). The Km and Vmax, were determined from the Lineweaver-Burk plot using the Microsoft Excel software.

### Effects of additives on LipM

The effects of twelve metal ions, the chelating agent ethylenediamine tetraacetic acid (EDTA), and two inhibitors Dithiothreitol (DTT), and β-mercaptoethanol (β-ME) on LipM were examined by adding at a final concentration of 1 or 10 mM for each reaction system. The residual activities of LipM were assayed in 50 mM Tris–HCl (pH 8.0) at 65 °C for 10 min using *p*-NP caprylate as substrate. Additionally, in order to inspect the organic solvent tolerance of LipM, the recombinant enzyme was incubated in 15 % (v/v, i.e., mixing 0.45 ml of organic solvent in 3 ml of the enzyme solution) or 30 % (v/v) of ten organic solvents at 65 °C with shaking speed of 150 rpm for 2 h. After that, tolerance of the enzyme against solvents was assayed under optimum conditions. Finally, in order to test the detergent tolerance of LipM, the purified recombinant enzyme was incubated in 0.05 % or 0.1 % (v/v) of eight commercial detergents at 65 °C for 30 min and then followed by residual activities of LipM assayed in under optimum conditions. All assays were conducted three times independently under the standard assay conditions (50 mM Tris–HCl, pH 8.0, at 65 °C for 10 min using *p*-NP caprylate as substrate), and the activity of the control with no additives was defined as 100 %. Values are means with standard error (SD) from three independent experiments.

### Nucleotide sequence accession number

The *lipM* nucleotide sequence and amino acid sequence have been submitted to the GenBank database under the accession numbers KF620114 and AHB29478, respectively.

### Application in selective hydrolysis of algal oil

The raw algal oil was analyzed by gas chromatograph-mass spectrometer (GC-MS) [15]. The purified LipM solution was lyophilized via a vacuum freeze. The total hydrolysis reaction system containing 3ml H_2_O, 1ml 50 mM Tris–HCl (pH 8.0), 1 g algal oil and 0.5 mg enzyme powder, with nitrogen purging for 1min, was then reacted at 60 °C with shaking speed of 200 rpm for 1–8 h. The methyl esters of raw algal oil and fatty acids in the glycerides were identified and quantified via an Agilent 7890A/5975C gas chromatograph equipped with a capillary column (DB-WAX, 30 m × 250 μm × 0.25μm). Additionally, the reacted mixtures in oil phase were analyzed by a thin layer chromatography (TLC, developing solvent: 85 % n-hexane, 15 % diethyl ether, 1 % acetic acid) and high performance liquid chromatography (HPLC, Detecter: ELSD 2000 ES, Alltech; column, Sepax HP-Silica, 4.6×250 mn) for detecting diacylglycerols (DAGs).

## Results

### Gene cloning, sequence analysis and molecular modeling

**Fig. 1:**
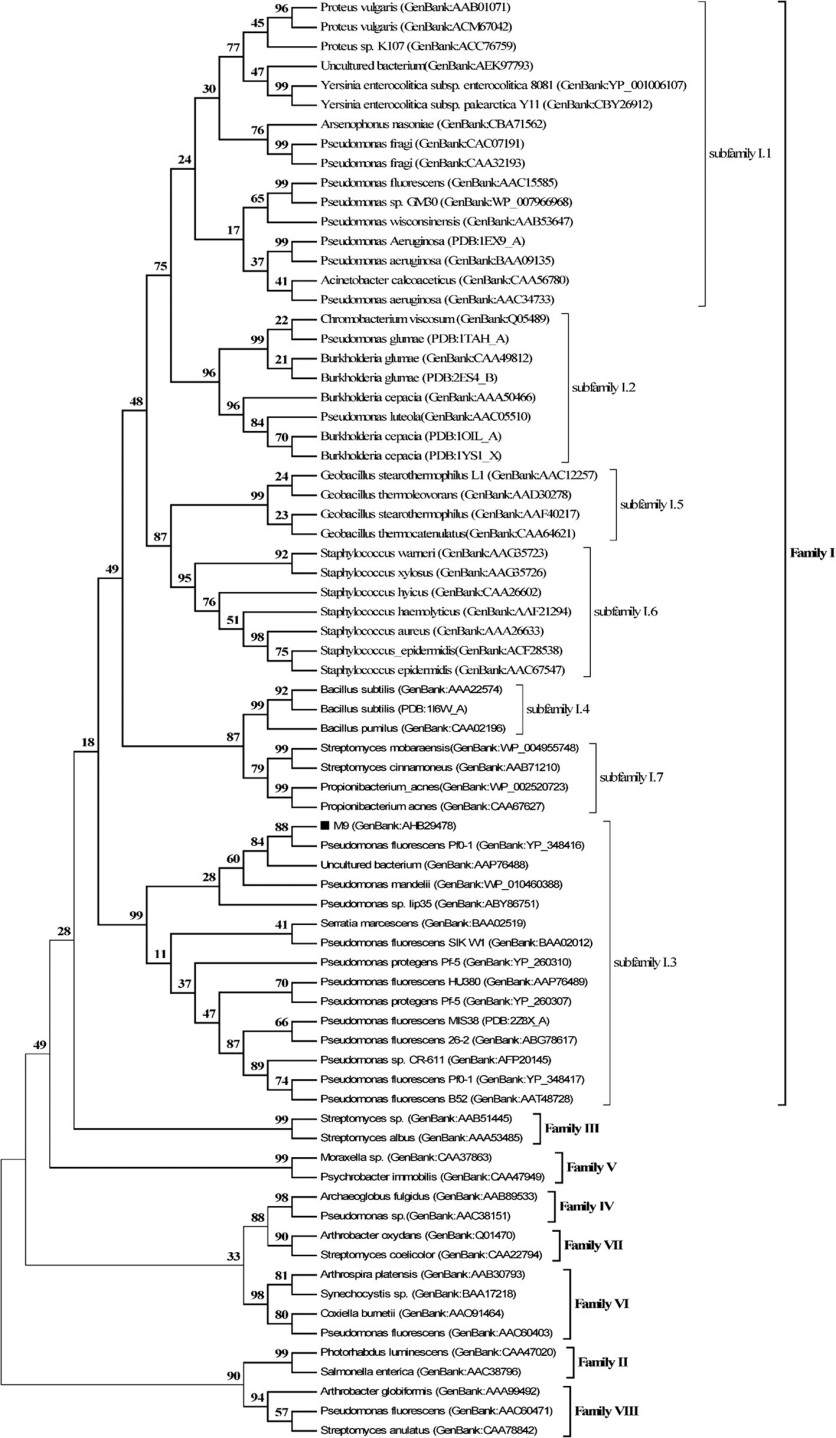
Phylogenetic analysis of LipM and other closely related lipases. The phylogenetic analysis was performed by the neighbor-joining method using MEGA 5.0. LipM was marked with a black square (■). The values at nodes indicate the bootstrap percentage of 1,000 replications. The lengths of the branches show the relative divergence among the reference lipase amino acid sequences and scale bar indicates the amino acid substitutions per position. Genbank accession numbers or PDB numbers are shown in brackets after each species name

A 1689 bp product was successfully obtained via touchdown PCR and genome walking. The amplified gene encoded a 562 amino acid polypeptide LipM with a predicted molecular mass of 58.7 kDa and a deduced *p*I of 4.63. Amino acid sequence alignment confirmed, LipM as a bacterial I.3 subfamily lipase (Fig. 1), with 82 % sequence identity with LipT from *P. mandelii* JR-1, and 54 % with PML from *Pseudomonas* sp. MIS38 [10] and Lip I.3 from *Pseudomonas* sp. CR-611 [11].

**Fig. 2:**
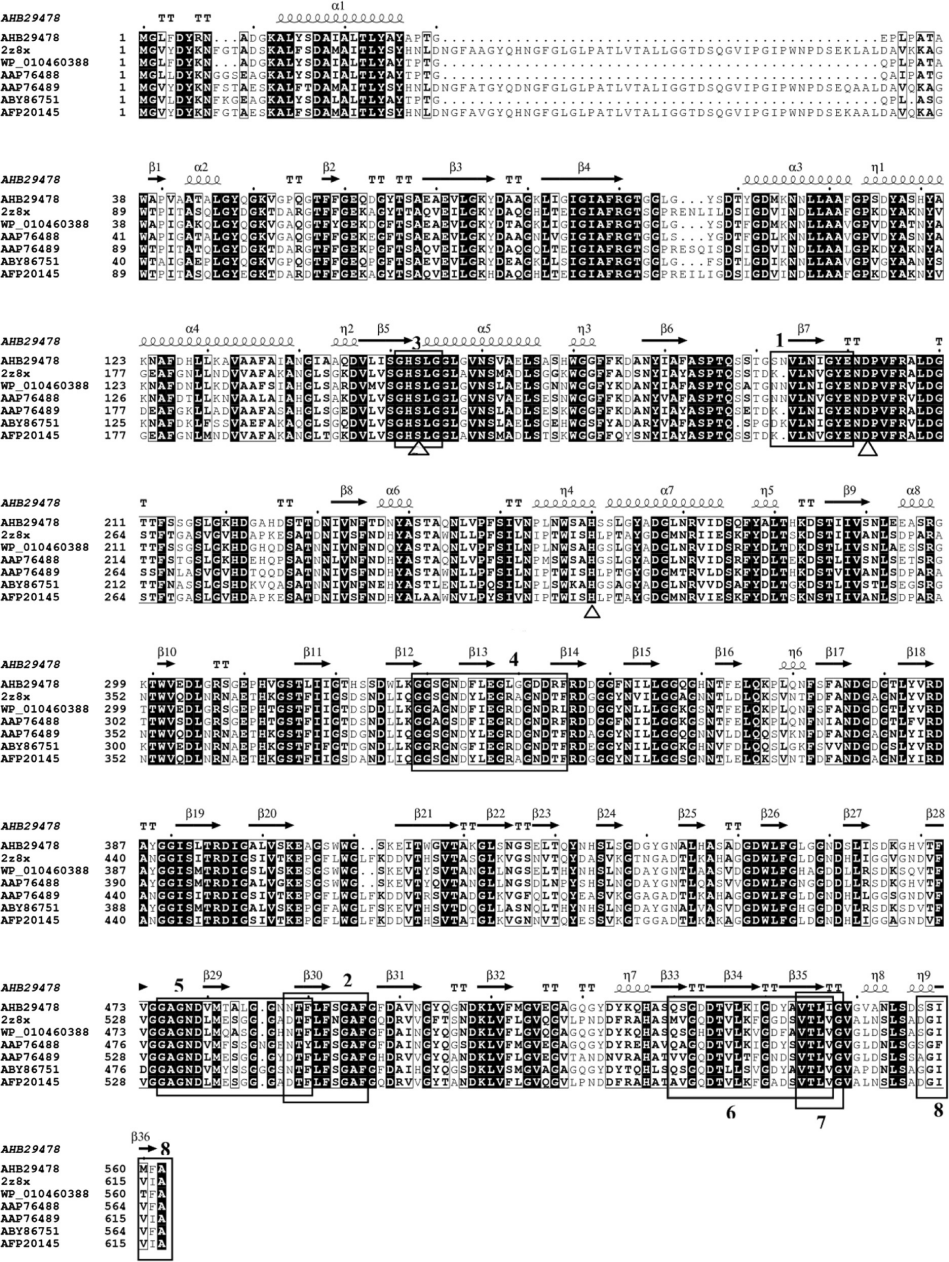
Multiple sequence alignment. **a** Multiple sequence alignment between LipM and other closely family I.3 Lipases: AHB29478, LipM from *Pseudomonas moraviensis* M9; 2z8x, PML from *Pseudomonas* sp. MIS38; WP_010460388, LipT from *Pseudomonas mandelii* JR-1; AAP76488 and AAP76489, LipA and LipB from uncultured bacterium; ABY86751, lip35 from *Pseudomonas* sp. lip35; AFP20145, Lip I.3 from *Pseudomonas* sp. CR-611. Empty triangles (△) represent putative catalytic residues at the corresponding positions of Ser_153_, Asp_202_ and His_260_. Sequence alignment was performed with Cluster 1.83 and visualized using ESpript 2.2. The alpha helix, beta sheet, random coil and beta turn are identical to α, β, η and T, respectively. 1: T5 primer; 2: T3 primer; 3: conserved catalytic motif; 4-5: the repetitive nine-residue motif GGXGXDXUX; 6: 18-residue amphipathic α-helix; 7: hydrophobic five-residue conserved motif; 8: four hydrophobic residues

The N-terminal domain of LipM lacks a signal sequence. Like other I.3 subfamily lipases [25, 26], the C-terminal domain contains four tandems nine-residue GGXGXDXUX motif repeats (Fig. 2 (4) and (5)), an 18-residue amphipathic sequence motif (Fig. 2 (6)), a hydrophobic six-residue conserved VTLIGV motif (Fig. 2 (7)) and a C-terminal SSIMFA motif (Fig. 2 (8)). These motifs were found to be relatively well conserved in type 1 secretion system passenger proteins [27].

**Fig. 3:**
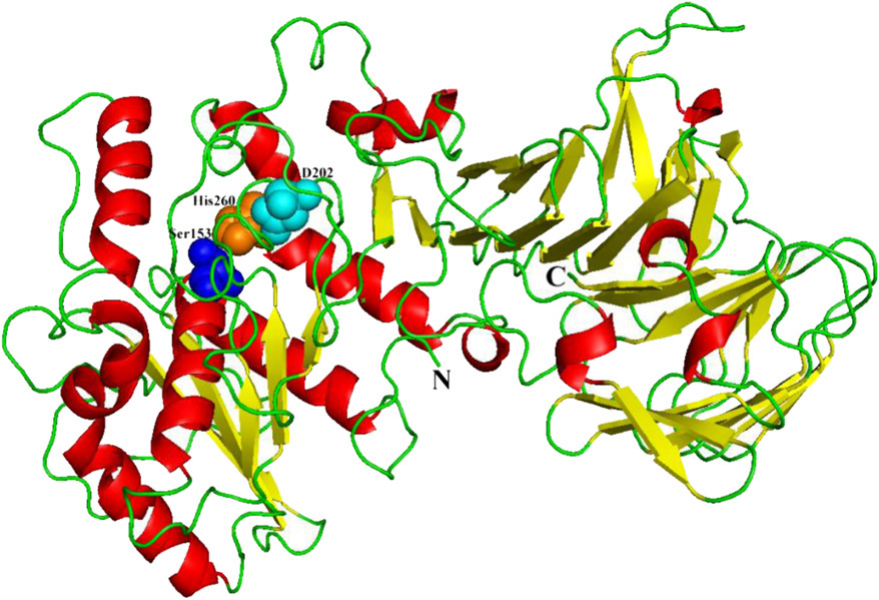
3D model of LipM. The α-helix, β-sheet, random coil and beta turn are shown cartoon in red, yellow and green, respectively. The catalytic triad (Ser_153_, Asp_202_ and His_260_) are shown as spheres in blue, cyan and orange, respectively. “**N**” and “**C**” denote the N and C termini, respectively

Automated molecular modeling of the 3D structure generated a homology model consisting of eight α-helices and 36 β-sheets that form the catalytic, α/β, and regulatory domains (Fig. 3). As validated by online PROCHECK (http://nihserver.mbi.ucla.edu/SAVES/), 88.8 % of the residues in the modeled structure are in the most favored regions, and only 7 out of 562 amino acids are in the disallowed regions. This result suggests that the model is satisfactory. Homology studies indicates that the catalytic domain is located at the N-terminal end of the polypeptide, while the C-terminal region forms the well-defined β-roll structure constituted by several antiparallel β-sheets that act as calcium-binding sites [25, 26].

### Expression and purification of LipM

**Fig. 4:**
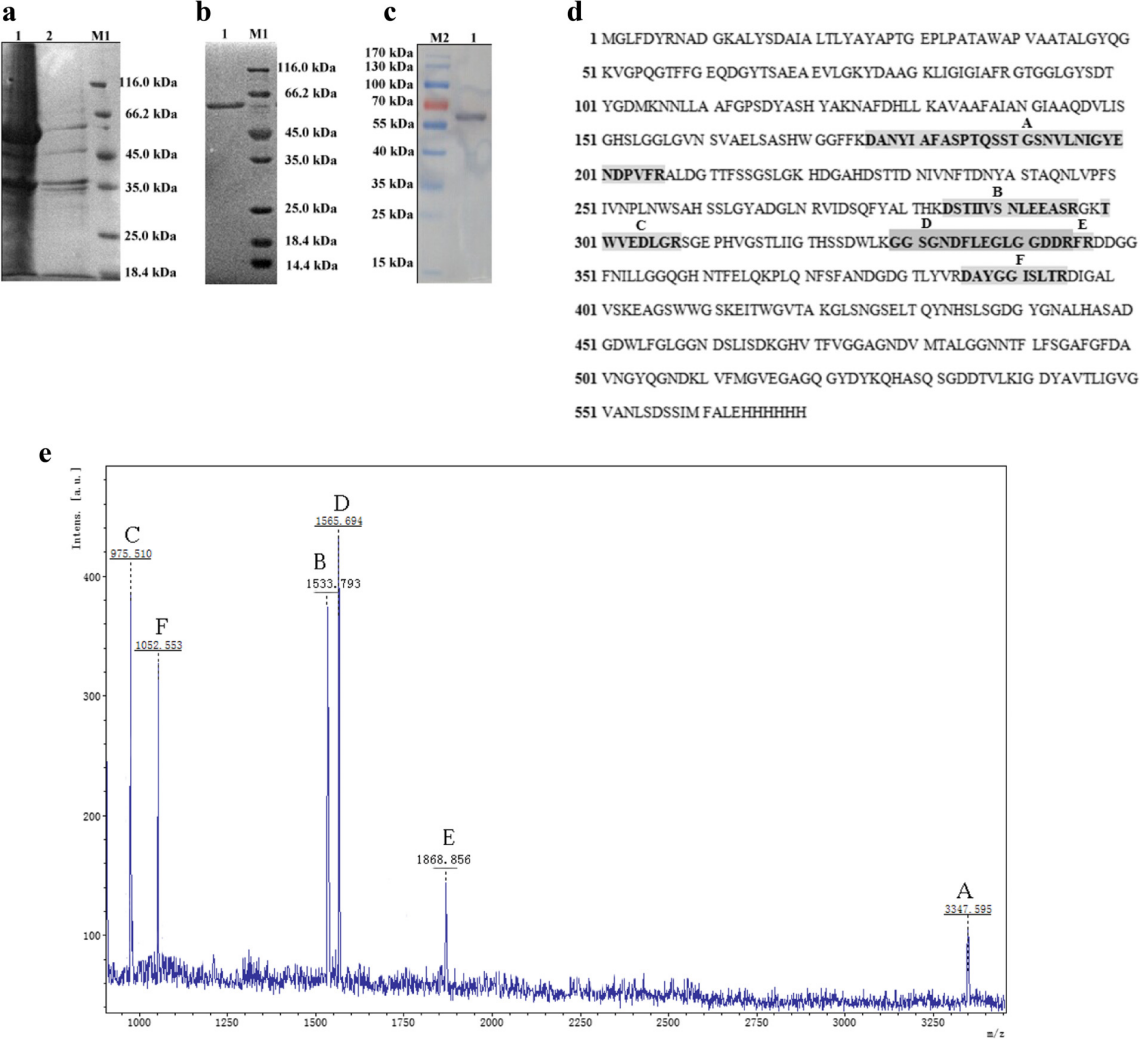
SDS-PAGE analysis of the purified LipM. **a** 1: inclusion bodies; 2: inclusion bodies solubilized in solubilization buffer; **b** 1: purified LipM; M1: unstained protein molecular weight markers (Fermentas, SM0431); **c** Western blot analysis of the purified LipM with an anti-His tag antibody. M2: prestained protein molecular weight marker (Fermentas, SM0671); 1: LipM combined anti-His tag primary antibody and horseradish peroxidase (HRP)-conjugated goat anti-mouse IgG second antibody; **d** Predicted lables (A-E) identify the peptide fragments of LipM whose molecular weights correspond to those of the peaks shown in the peptide mass fingerprint; **e** Peptide mass fingerprint generated by MALDI-TOF mass spectrometry of the purified LipM (x-axis, m/z ratio; y-axis, species abundance in terms of percent signal intensity)

After induction with 0.2 mM IPTG at 16 °C for 20 h, LipM was expressed predominantly in inclusion bodies in *E. coli* BL21 (DE3). Protein was dialyzed and purified by Ni-NTA affinity chromatography as previously described [10] with minor modifications. LipM was purified 2.34-fold and 8.8 mg of protein per litre of culture was prepared which equated to a recovery rate of 40.43 % (Table 2). LipM was present in inclusion bodies (Fig. 4a), and was solublized in 6 M guanidine HCl solubilization buffer and purified by Ni-NTA affinity chromatography. A single band with a molecular weight of approximately 60 kDa was presented following separation by sodium dodecyl sulfate polyacrylamide gel electrophoresis (Fig. 4b) and western blotting (Fig. 4c), which coincides well with the theoretic molecular mass of LipM. Additionally, peptide mass fingerprinting (Fig. 4e) of this single band confirmed that it was the predicted lipase (Fig. 4d) encoded by the putative 1689 bp ORF.

**Table 2:** Purification of LipM from *Escherichia coli* BL21 (DE3)^a^

	Concentration (mg/ml)	Volume (ml)	Total protein (mg)	Specific activity (U/mg)	Purification fold	Recovery (%)
Dialysis	1.02	50	50.9	60.6	1	100
LipM	0.44	20	8.8	141.73	2.34	40.43

^a^Lipase activity was determined with *p*-nitrophenyl caprylate as substrate

### Substrate specificity

**Fig. 5:**
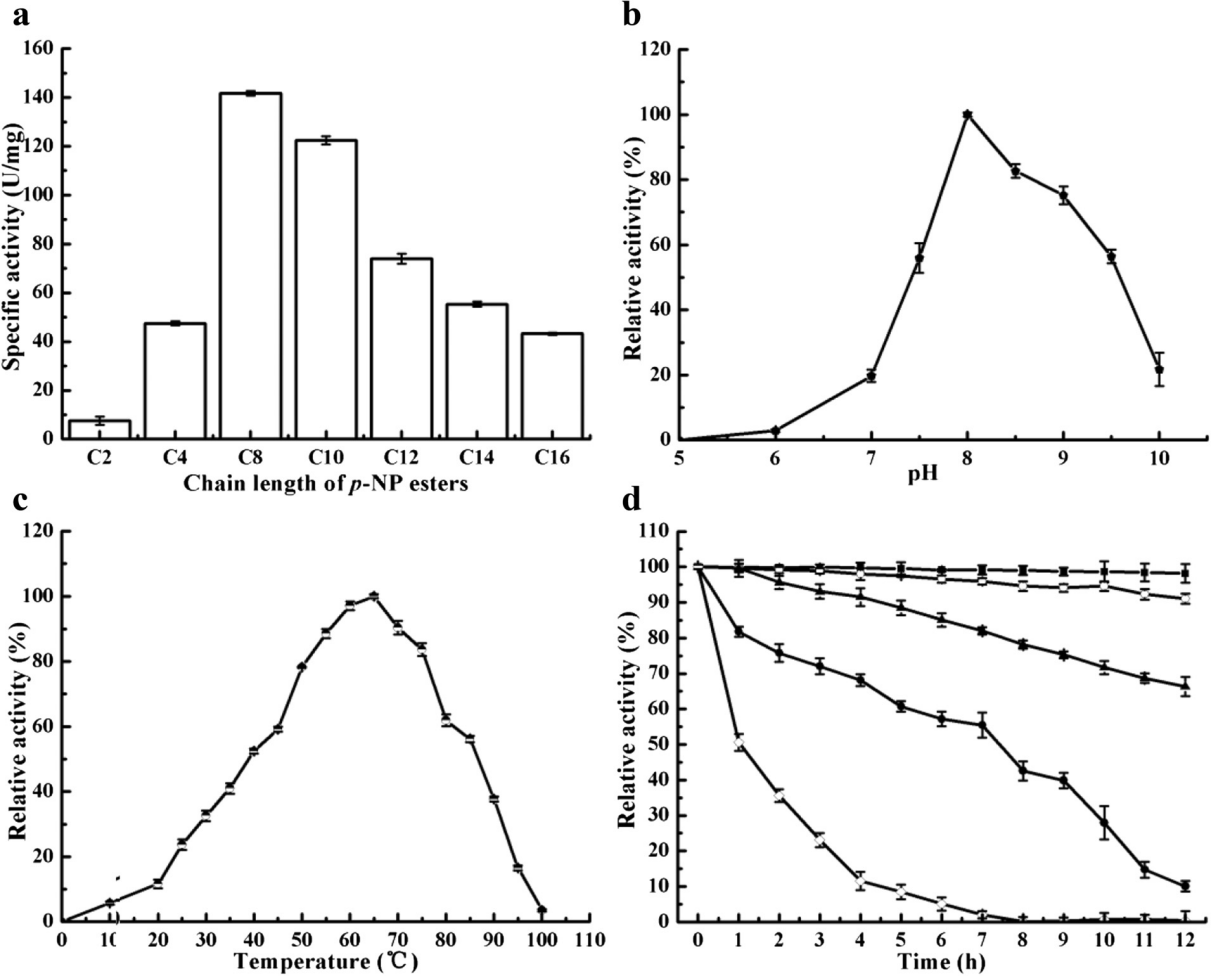
Characterization of LipM and effects of additives on LipM. **a** Specific activities of LipM towards *p*-NP esters of various chain lengths (C2, acetate; C4, butyrate; C8, caprylate; C10, decanoate; C12, laurate; C14, myristate; and C16, palmitate); **b** The pH profile of LipM. These relative activities of LipM were measured at different pH values. The enzyme activity in Tris-HCl (50 mM, pH 8.0) was taken as 100 %. Buffers used (final concentration 50 mM) were citrate-phosphate buffer (pH 4.0–6.5), Tris–HCl buffer (pH 7.0–8.5), and Glycine-NaOH buffer (pH 9.0–10.0); **c** Effect of temperature on the activity of LipR; **d** Thermal stability of LipM. The enzyme was incubated at 60 °C (■), 65 °C (○), 70 °C (▲), 75 °C (●) and 85 °C (◊) and for the indicated time

The purified LipM possessed the ability to hydrolyze *p*-nitrophenyl (NP) esters with fatty acyl chains ranging from C2 to C16 (Fig. 5a), and exhibited maximum lipase activity towards *p*-NP caprylate (100 %, 141.73 U mg^−1^). Compared with *p*-NP caprylate (C8), the relative activities of LipM towards other substrates were as follows: *p*-NP acetate (C2) was 5.39 %, *p*-NP butyrate (C4) 33.5 %, *p*-NP caprate (C10) 86.35 %, *p*-NP laurate (C12) 52.21 %, *p*-NP myristate (C14) 39.02 %, and *p*-NP palmitate (C16) 30.05 %. LipM therefore showed higher activity towards medium/long chain substrates (C8, C10, C12, C14, and C16). Additionally, the activity of LipM towards olive oil was 238.6 U/mg^−1^.

### Effects of pH and temperature on enzyme activity

The recombinant LipM exhibited maximum activity towards *p*-NP caprylate at pH 8.0 (Fig. 5b) and retained more than 55 % of activity between pH 7.5 to 9.5. However, at a pH less than 6 more than 90 % of activity was lost, suggesting that it is an alkaline lipase. LipM displayed maximal activity at 65 °C (Fig. 5c), and retained over 50 % of activity between 40–85 °C, with 16.5 % at 95 °C, 11.7 % at 20 °C and 5.8 % at 10 °C (Fig. 5c). These results suggest that LipM is a eurythermic lipase that functions well across a wide temperature range. Additionally, *p*-NP caprylate was the preferred substrate, with *K*_m_ of 0.36 mM and a *k*_cat_ of 35.7 s^−1^, and a catalytic efficiency (*k*_cat_/*K*_m_) of 99.17 s^−1^ mM^−1^.

The thermostability of LipM was studied in Tris–HCl (pH 8.0) at 60 °C, 65 °C, 70 °C, 75 °C and 85 °C for 12 h (Fig. 5d). After incubation for 12 h at 60 °C and 65 °C, LipM retained over 90 % of its original activity. Incubation at 70 °C and 75 °C for 5 h resulted in over 60 % of maximal activity and 35.5 % of activity was remained after exposure at 85 °C for 2 h, indicating a very high thermostability. However, it was inactivated when incubated at 75 °C and 85 °C in a pseudo-first-order manner, with *t*_1/2_ values of 435 min and 60 min, respectively.

### Effects of metal ions, inhibitors, organic solvents and detergents

**Fig. 6:**
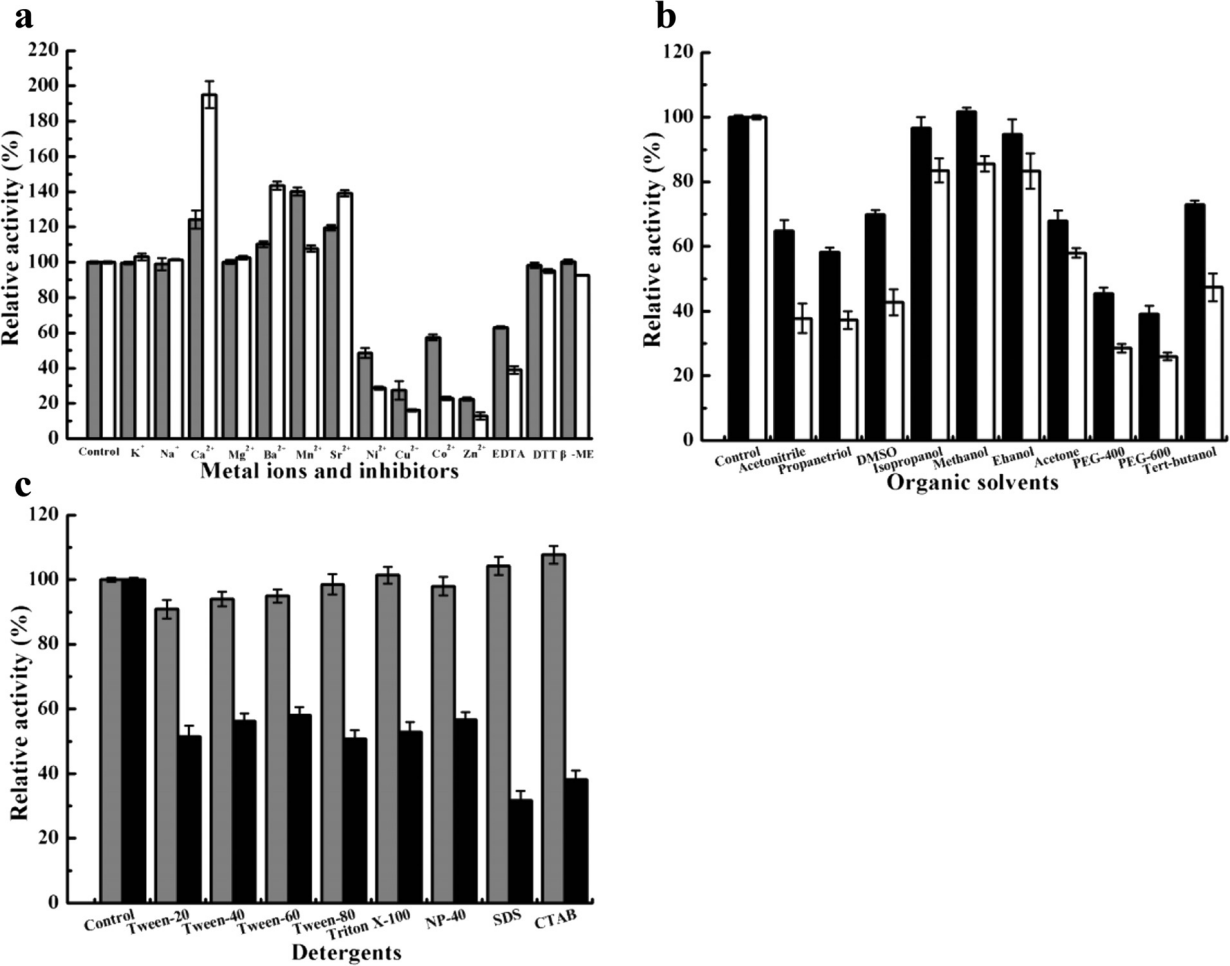
Effects of additives on LipM activity. **a** effects of different metal ions and inhibitors on LipM activity, measured at 1 mM (grey bars) and 10 mM (white bars) final concentrations; **b** effects of some organic solvents on LipM activity, tested at 15 % (black bars) and 30 % (white bars) final concentrations; **c** effects of some detergents on LipM activity, studied at 0.05 % (grey bars) and 0.1 % (black bars) final concentrations. The residual activity was measured by a standard assay. The values represent the means of three independent experiments (Mean ± standard error)

At a final concentration of 1 mM or 10 mM, LipM was enhanced by Ca^2+^ (1.35-fold; 2.08-fold), Ba^2+^ (1.10-fold; 1.43-fold), Mn^2+^ (1.40-fold; 1.07-fold) and Sr^2+^ (1.20-fold; 1.39-fold), respectively (Fig. 6a). However, LipM was fully inhibited by Fe^2+^ and strongly inhibited by Ni^2+^ (48.55 %; 28.75 %), Cu^2+^ (27.39 %; 16.11 %), Co^2+^ (57.41 %; 22.83 %), Zn^2+^ (22.49 %; 12.84 %), and EDTA (62.99 %; 39.02 %). Strong activation by Ca^2+^ is consistent with the predicted calcium-binding sites [25, 26]. In contrast, Mg^2+^, K^+^, Na^+^, DTT and β-ME had little or no effect on the catalytic activity of LipM at 1 mM or 10 mM. LipM contains no cysteine residues, consistent with the lack of any effect on activity with the known reducing agents DTT and β-ME, which can reduce the disulfide bonds of proteins and prevent the formation of intra- or intermolecular disulfide bonds between cysteine residues [1, 22]. Both DTT and β-ME had almost no effect on LipM, which proves that LipM is a lipase without cysteine residues.

LipM activity was decreased to some extent when treated with 15 % (v/v) or 30 % (v/v) organic solvents (Fig. 6b). Specifically, activity was decreased by more than 40 % in a mixture of 15 % (v/v) acetonitrile, propanetriol, DMSO, acetone, *tert*-butanol, PEG-400 and PEG-600. When the concentration of the aforementioned organic solvents was increased to 30 % (v/v), LipM activity was strongly inhibited. Comparatively, 15 % (v/v) isopropanol, methanol and ethanol had little or no effect on LipM, and even 30 % (v/v), more than 80 % of activity remained. High stability and activity in organic solvents are major advantages for the industrial application of enzymes [2, 8], and of bacterial lipases in particular. The outstanding stability of LipM in isopropanol, methanol and ethanol may therefore prove useful for use in biocatalysis such as optical resolution of chiral compounds, organic synthesis [1, 28].

Detergents affected the activity of LipM at concentrations of 0.05 % or 0.1 % (Fig. 6c). LipM retained over 90 % of its initial activity in the presence of 0.05 % detergents, even with ionic detergents SDS (anions) and CTAB (cationic), the activities of LipM was 1.04-fold and 1.07-fold of the initial one. When the concentration of detergents was increased to 0.1 %, LipM retained 30–40 % of its original activity in the presence of the ionic detergents SDS and CTAB, and 60 % in the presence of the non-ionic detergents Tween-40, Tween-60, Triton X-100 and NP-40, which is reminiscent of the lipase SAL-PP1 from *Staphylococcus aureus* [5]. Moreover, due to higher tolerance to non-ionic than ionic detergents, LipM may have potential for use in the detergents industry.

### Selective hydrolysis of algal oil to enrich PUFAs

**Fig. 7:**
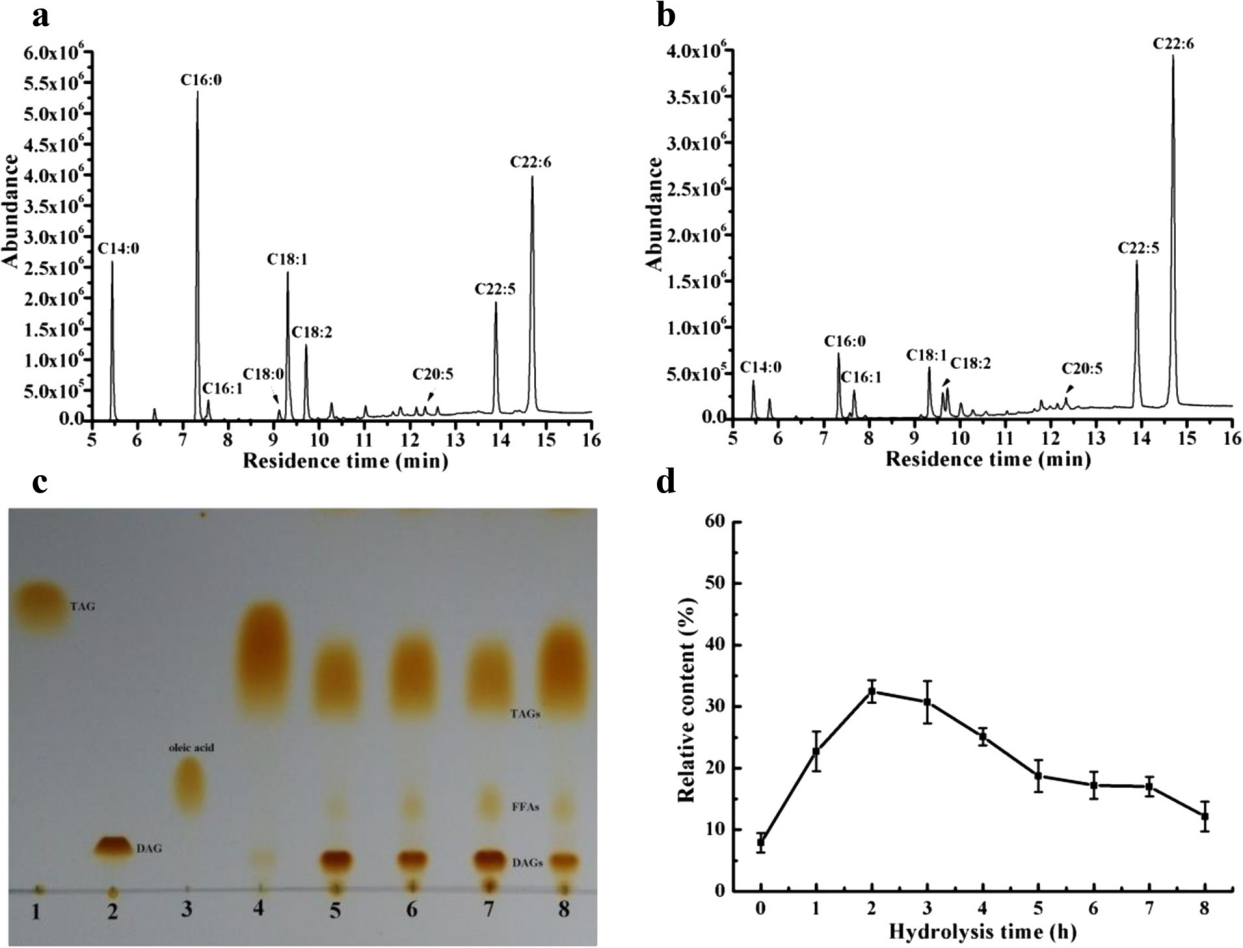
Selective hydrolysis of algal oil by LipM. **a** The gas chromatograph-mass spectrometry (GC–MS) analysis of raw algal oil showed kinds of fatty acid methyl esters. C14:0, methyl tetradecanoate; C16:0, hexadecanoic acid, methyl ester; C16:1, 9-hexadecenoic acid, methyl ester; C18:0, methyl stearate; C18:1, 9-Octadecenoic acid, methyl ester; C18:2, 9,12-Octadecadienoic acid, methyl ester; C20:5, 5,8,11,14, 17-Eicosapentaenoic acid, methyl ester; C22:5, 4,7,10,13,16-docosapentaenoate, methyl ester; C22:6, 4,7,10,13,16,19-docosahexaenoate, methyl ester; **b** The GC–MS analysis of algal oil showed kinds of fatty acid methyl esters after hydrolyzing for 3 h. **c** Thin layer chromatography (TLC) analysis after hydrolysis algal oil of LipM. 1:TGA (standard sample); 2:DGA (standard sample); 3: oleic acid (standard sample); 4:raw algal oil; 5–8: algal oil was hydrolyzed to remained partially triacylglycerols (TAGs), produce corresponding diacylglycerols (DAGs) and free fatty acids (FFAs) at 1 h, 2 h, 3 h and 4 h. **d** According to the analysis of HPLC, the relative contents of produced diacylglycerols (DAGs, ■) after hydrolyzing different time

According to gas chromatography-mass spectrometry (GC-MS) analysis (Fig. 7a), algal oil contains 14:0 (tetradecanoic acid, 10.94 %), 16:0 (hexadecanoic acid, 23.95 %), 16:1 (hexadecenoic acid, 1.8 %) 18:0 (stearic acid, 0.76 %), 18:1 (octadecenoic acid, 12.46 %), 18:2 (octadecadienonic acid, 6.03 %), 20:5 (eicosapentaenoic acid, EPA, 0.74 %), 22:5 (docosapentaenoic acid, DPA, 10.41 %), and 22:6 (docosahexaenoic acid, DHA, 27.79 %). Approximately 94.88 % of algal oil in the form of triacylglycerols (TAGs), and the PUFAs EPA, DPA and DHA account for 38.94 %, suggesting it may be suitable for PUFAs enrichment.

GC-MS analysis of algal oils following hydrolysis by LipM showed that medium chain (C14 and C16) fatty acids were released prior to long chain (C18) and/or polyunsaturated fatty acids (≥ C20). After 3 h of hydrolysis, the relative content of EPA, DPA and DHA in the derivative glycerides increased to 1.25 %, 17.61 %, and 47.02 %, respectively (Fig. 7b). This accounted for 65.88 %, which was much higher than the PUFA content (38.94 %) of algal oil before hydrolysis with LipM. The above result also indicates that LipM has strong substrate specificity for particular fatty acids.

In addition, TLC analysis revealed that hydrolysis of algal oil by LipM produced diacylglycerols (DAGs) and free fatty acids (FFAs), but some TAGs remained (Fig. 7c). HPLC analysis demonstrated that the raw algal oil contained 7.9 % DAGs (data not shown), while the relative content of DAGs in the derived glycerides was increased substantially during the first 2 h of hydrolysis, and peaked at 32.46 % before gradually decreasing 30.73 % between 3–8 h (Fig. 7d).

## Discussion

Touchdown PCR offers a simple and rapid means to optimize PCR approaches, without the need for lengthy optimizations and/or redesigning of primers [29]. Together with the genome walking method, Touchdown PCR was used successfully to amplify *lipA* from *Acinetobacter* sp. XMZ-26 [22] and *lip* I.3 from *Pseudomonas* CR-611 [11]. So far, the genome of *P. moravensis* has not been published, and thus potential lipase genes were cloned using primers based on conserved motifs. In general, degenerate primers were designed based on the sequences upstream and downstream of the conserved GHSLG motif of subfamily I.3 lipases [27]. Upon close inspection of sequence alignment data, the SNVLNIGYE and NTFLFSGAF motifs (Fig. 2 (1) and (2)) were also found to be conserved among subfamily I.3 lipases, and this knowledge was used to design degenerate primers T5 and T3. Till now, there are no relative reports on the design degenerate primers for the two regions. Touchdown PCR and genomic walking using these primers resulted in amplification of the full-length *lipM*. Thus, the two different domains can be used for degenerate primer design for amplification of subfamily I.3 lipase genes in future work. Additionally, we showed that Touchdown PCR and genome walking approaches can be effective for cloning genes from organisms whose genome sequences are not available.

Most industry processes are performed at temperatures higher than 50 °C, and thermostable enzymes are of great importance [1, 28]. Even thermostable lipases (see Table 3) lose activity to some extents during long incubations at temperatures exceeding 60 °C. The results of the present study showed that LipM is unusually highly thermostable, indicating great potential use in various industries including fat and oil modification, cosmetics, pharmaceuticals, organic synthesis, oleo-chemicals and biodiesel production [6]. Lipases with a broad temperature range have been reported, including EH28 that has a temperature range of 30 °C–80 °C [28], LipZ01 (20 °C–80 °C) [24], SAL-PP1 (10 °C–65 °C) [5]. Interestingly, LipM displays an even a broader temperature range of from (10 °C–95 °C) and is one of the most eurythermic lipases ever discovered. This indicates that LipM has great potential for various high-temperature industrial applications such as the removal of pitch from pulp in the paper industry, the removal of subcutaneous fat in the leather industry [30], as a medium-temperature catalyst in the enrichment of PUFAs [15], and as a low-temperature catalyst in the food or detergent industries [12].

**Table 3:** Characterization on thermostability of some bacterial lipases

Microorganism	Optimum temperature (°C)	Incubation conditions (°C, h)	Residual activity (%)	Authors
*Pseudomonas* sp. MIS 38	30	NM	NM	Amada et al.
*Pseudomonas mandelii* JR-1	25	NM	NM	Kim and Lee
*Pseudomonas* CR-611	30	30, 1	60	Panizza et al.
		60, 1	0	
*Pseudomonas moraviensis* M9	65	60, 12	98.2	This study
		65, 12	91.0	
		70, 12	66.3	
		75, 5	60.7	
		85, 2	35.5	
*Acinetobacter* sp. EH28	50	60, 1.25	80-90	Ahmed et al.
		70, 1	50-60	
*Aneurinibacillus thermoaerophilus* HZ	65	60, 4	70-80	Masomian et al.
		65, 3	50-60	
		70, 1.5	40-50	
*Bacillus coagulans* BTS-3	55	55, 2	50	Kumar et al.
		60, 0.5	50	
		70, 0.3	75	
*Bacillus thermoleovorans* CCR11	60	50/60, 1	75	Castro-Ochoa 2et al.
		70, 1	0	
*Burkholderia cepacia* ATCC 25416	60	50, 1	85	
		60, 1	80	Wang et al.
		70, 1	59	
*Burkholderia multivorans* PSU-AH130	55	55, 3	98	Chaiyaso et al.
		65, 2	50	
		65, 3	44	
Metagenomic library	40	50.7, 1	94.0	Glogauer et al.
		60.6, 1	75.8	
		70, 1	64.9	
		80, 1	31.4	
Metagenomic library	50	60, 1 70, 1	53 33	Faoro et al.
*Staphylococcus aureus*	55	60, 1	65.3	Sarkar et al.
		70, 1	62.62	
		80, 1	12.62	

*NM* not mentioned

Lipases are diverse in their sensitivity to solvents [28], and their stability in the presence of organic solvents is a requisite property when used in non-aqueous systems [22]. In this study, lipase LipM from *P. moraviensis* M9 exhibited fair stability in the presence of 15 % and 30 % of different organic solvents, as the decrease in activity of lipase was obvious in the presence of most of the organic solvents. Similar result was observed by Sarker [5] for alkali-thermostable lipase from *S. aureus*. This phenomenon may be due to the reduction of water activity around the protein molecules promoting structural denaturation [1, 8]. With the treatment of 30 % of ethanol, methanol, and isopropyl alcohol, LipM retained almost the same or higher relative activity with that of the lipase from *Acinetobacter* EH28 [28]. However, organic solvent tolerance of LipM incubating at 65 °C for 2 h was obviously stronger than the lipase from *Acinetobacter* EH28 incubating at 50 °C for 1 h [28], LipA from *B. cepacia* ATCC 25416 incubating at 37 °C for 2 h [8], and SAL-PP1 from *S. aureus* incubating at 37 °C for 90 min [5]. Thus, it can be seen that LipM has greater potential than the latter three lipases in application in organic synthetic industry and transesterification reaction. Additionally, 30 % of acetone could increase the activity of LipA from *B. cepacia* ATCC 25416 [8], 30 % of DMSO and acetone had stimulatory effect on the activity of lipase from *Acinetobacter* EH28 [28], 30 % of DMSO and methanol could also activated the lipase from *Aneurinibacillus thermoaerophilus* strain HZ [1] and LipA from *Acinetobacter* sp. XMZ-26 [22], while there was no stimulation effect on LipM by the above mentioned solvents. It is well known that the effect of organic solvents on enzyme activity differs from lipase to lipase [1]. These results can be generally explained that different solvents may induce some different structural changes in enzymes and cause various degree of disaggregation of lipases, resulting in varying residual activities [28]. Moreover, there is no clear correlation between the solubility of an organic solvent in water and the stability of lipase in its presence [1].

Current research on the enrichment of PUFAs suggests more wore is certainly needed (Table 4), although there has been some success. Immobilized *Rhizopus japonicas* lipase enriched DHA in soybean oil from 18 % to 25 % (an increase of 0.39-fold) after reacting for 24 h [19]. Similarly, hydrolysis of sardine oil by lipase from *Aspergillus niger* and *Mucor javanicus* for 3 h increased DHA from 13.63 % to 18.72 % (0.35-fold) and 13.62 % to 21.34 % (0.57-fold), respectively [16], while a 4 h hydrolysis of oil from *Chlorella protothecoides* with immobilized YlLIP2 increased DHA content from 19.32 % to 31.53 % (0.63 fold) [15]. In the present study, hydrolysis of algal oil by LipM for 3 h increased DHA from 27.79 % up to 47.02 % (0.69-fold) and the total percentage of PUFAs (EPA, DPA, and DHA) from 38.94 % to 65.88 % (Table 4). LipM therefore appears to be superior for DHA enrichment and the simultaneous enrichment of EPA and DPA from algal oil indicates great potential in PUFAs enrichment.

**Table 4:** Characterization of some lipases for enriching PUFAs

Microorganism	Relative content in raw oil (%)	Reaction time (h)	Relative content in derived glycerides (%)	Authors
*Pseudomonas moraviensis* M9				This study
DHA	27.79	3	47.02	
EPA, DPA and DHA	38.94	3	65.88	
*Rhizopus japonicas*				Khare and Nakajima
DHA	18	24	25	
*Aspergillus niger*				Okada and Morrissey
DHA	13.63	3	18.72	
*Mucor javanicus*				Okada and Morrissey
DHA	13.62	3	21.34	
*Yarrowia lipolytica*				Yan et al.
DHA	19.32	4	31.53	
*Geotrichum candidum*				Shimada et al.
EPA and DHA	38.5	16	48.7	
*Candida rugosa*				McNeill et al.
EPA and DHA	30	24	45	
*Psedomonas fluorescence*				Pawongrat et al.
EPA and DHA	32.1	24	56	

DAGs consist of diesters of glycerol and contain at least 90 % acylglycerols, and are therefore classified as non-ionic surfactants [31]. Unlike ionic tensoactive agents, DAGs show no side effects whenever ingested or applied to skin [32], and can be widely used in the food, pharmaceutical, and cosmetic industries [31]. Hydrolysis of algal oil by LipM for 2–3 h increased the percentage of DAGs in the derived glycerides. To our knowledge, this is the first report of such an activity in combination with the simultaneous enrichment of EPA, DPA and DHA, meanwhile with increase of DAGs in the derivative glycerides. Therefore, LipM possess great potential prospect in future industrial applications.

## Conclusion

This study identified and isolated a novel subfamily I.3 lipase from *P. moraviensis* M9. Detailed biochemical characterization revealed LipM to be unusually eurythermic and thermostable, and medium tolerant to organic solvents and detergents. In addition, LipM was particularly effective for enriching PUFAs, indicating potential as a biocatalyst for future food industrial applications.

## Abbreviations

EDTA: Ethylenediamine tetraacetic acid
DHA: Docosahexaenoic acid
PUFAs: Polyunsaturated fatty acids
EPA: Eicosapentaenoic acid
DPA: Docosapentaenoic acid
GuHCl: Guanidine hydrochloride
ORF: Open reading frame
DTT: Dithiothreitol
β-ME: β-mercaptoethanol
DMSO: Dimethylsulfoxide
TBA: Tert butyl alcohol
SDS: Sodium Dodecyl Sulfate
CTAB: Cetyltrimethyl Ammonium Bromide
NP-40: Nonyl phenoxypolyethoxylethanol 40
GC-MS: Gas chromatography-mass spectrometry
TAGs: Triacylglycerols
DAGs: Diacylglycerols
FFAs: Free fatty acids
IPTG: Isopropyl-β-D-1-thiolgalactopyranoside
LB: Luria Bertani
Ni-NTA: Nickel-nitrilotriacetic acid
BSA: Bovine serum albumin
SDS-PAGE: Sodium dodecyl sulphate-polyacrylamide gel electrophoresis
PVDF: Polyvinylidene fluoride
HRP: Horseradish peroxidase
DAB: Diaminobenzidine
TLC: Thin layer chromatography
HPLC: High performance liquid chromatography
